# Community factors related to healthy eating & active living in counties with lower than expected adult obesity rates

**DOI:** 10.1186/s40608-016-0129-x

**Published:** 2016-11-18

**Authors:** Maureen E. Canavan, Emily Cherlin, Stephanie Boegeman, Elizabeth H. Bradley, Kristina M. Talbert-Slagle

**Affiliations:** 1 Department of Health Policy and Management, Yale University School of Public Health, 60 College St, New Haven, 06520 CT USA; 2 Yale Global Health Leadership Institute, 2 Church Street South, Suite 409, New Haven, 06529 CT USA

**Keywords:** Organizational factors, Community dynamics, Strategic thinking

## Abstract

**Background:**

Adult obesity rates in the United States have reached epidemic proportions, yet vary considerably across states and counties. We sought to explore community-level factors that may be associated with reduced adult obesity rates at the county level.

**Methods:**

We identified six U.S. counties that were positive deviants for adult obesity and conducted semi-structured interviews with community leaders and government officials involved in efforts to promote healthier lifestyles. Using site visits and in-depth qualitative interviews, we identified several recurrent themes and strategies.

**Results:**

Participants: 1) developed a nuanced understanding of their communities; 2) recognized the complex nature of obesity, and 3) implemented a county-wide strategic approach for promoting healthy living. This county-wide approachwas used to a) break down silos and build partnerships, b) access community resources and connections, and c) transfer ownership to community members.

**Conclusions:**

We found that county leaders focused on establishing a county-wide structure to connect and support community-led initiatives to promote healthy living, reduce obesity, and foster sustainability. Findings from this study can help inform county-level efforts to improve healthy living and combat the multi-faceted challenges of adult obesity across the U.S.

## Background

Obesity has reached epidemic proportions in the United States (U.S.), with one out of every four adults having a body mass index (BMI), ≥ 30 [1, 2]. Individuals who are obese are more likely to develop chronic diseases [3–6], and as of 2009, the annual cost of obesity in the U.S. was approximately 300 billion dollars [7]. Obesity rates vary considerably across individual states [8] and counties [9, 10]. Such geographic variation raises questions about the influence of local, community, and regional factors on obesity.

Multiple studies describe efforts to address obesity: for example for individuals [11–15], schools [16, 17], worksites [18, 19], and the built environment and community norms [20, 21]. However, these programs and initiatives to think beyond the individual level have focused more on organizations [22, 23], with less available literature on county-level experiences. Considerable variation exists not only in the types of obesity intervention programs implemented, but also in measures of their effectiveness, making it difficult to know whether and how such programs work relative to each other, and which may work best for a given population. One strategy for identifying effective health interventions is to study members of a group that are performing better than expected. Such better-than-expected performers, called positive deviants, may exhibit characteristics or approaches that could also be effective when generalized to the larger population [24]. This approach has been used to address hand hygiene compliance [25], diabetes management [26], timeliness of cardiology care [27], hospital readmission [28], and adolescent pregnancies [29]. To our knowledge, however, no study has examined county-level positive deviants for adult obesity rates.

Accordingly, our research aim was to identify positive deviant counties for obesity and using semi-structured in-depth interviews to determine any community level factors that were present across all counties that might give insights into how the lower rates of obesity were achieved by these counties. Findings from this inquiry may be useful to other localities as they consider strategies for reducing obesity rates. We identified a set of six counties with particularly low obesity rates despite socioeconomic profiles that would predict higher obesity rates and conducted in-depth, qualitative interviews with 80 individuals across the six counties. Our findings may suggest avenues for interventions to counter the ongoing challenge of obesity in the U.S.

## Methods

### Study design and sample

To identify positive deviant counties with lower than expected obesity rates, we used a dataset of all 3141 county and county level equivalents in the U.S. from the 2013 Robert Wood Johnson County Health Rankings dataset [30]. Based on our research, the sociodemographic variables most strongly correlated with county obesity rates were education, income, and race/ethnicity. Thus, to identify counties at elevated risk for obesity, we selected counties marked by all three of these characteristics: 1) a lower percent of adults than average nationally who had completed high school or high school equivalence, 2) median household incomes lower than the national median household income, and 3) a greater percent of adults compared with national averages from minority race/ethnicity (black or Hispanic). From this set of at risk counties (*N* = 3141), we selected counties that had obesity rates (where obesity was defined as BMI < 30) that were in the lowest quartile nationally and yet that were located in a state with the higher than the national average in obesity rates. Because the resulting counties were located in states with high obesity and at the county-level had socioeconomic risk characteristic for higher obesity and yet had relatively low obesity rates, these counties were considered positive deviants. We first identified the 15 counties that met our criteria for positive deviants and then selected six counties that were representative of regional, geographic, and sociodemographic features of the larger sample. We started with counties with the lowest obesity rates and those from different states. We stopped visiting new counties and conducting additional interviews after reaching theoretical saturation.

In those six positive deviant counties, we searched websites and identified the head of the health department (or equivalent entity) and contacted those individuals with requests to suggest community leaders who were involved in programs, initiatives, and other activities that promoted healthy eating and active living lifestyles. The resultant list of potential interviewees contained individuals who worked within health departments, local non-profit organizations, faith-based organizations, local hospitals, and other community programs. We contacted those individuals, requested interviews, and also identified additional participants from each county using the snowball sampling method. Across the six counties we interviewed 80 individuals for an average of 12 interviewees per county; however, in some cases, people requested to be interviewed together, which we accommodated as watching their interactions and teamwork was also helpful in understanding how they conducted their work. (Of the 80 participants, approximately 20 were interviewed in 2–4 person groups).

### Data collection

All interviews were conducted using a semi-structured interview guide with follow-up questions and prompts in order to elicit additional detail and utilized established approaches to enhance the rigor of our findings [31–34] (Table 1). Interviews lasted between 45 and 75 min and focused on participants’ involvement in activities or programming intended to promote a healthy lifestyle within the county as well as their interaction with county residents. On average, interviews lasted 1 h and were conducted in the summer and fall of 2014. All interviews were audio-recorded after obtaining consent, independently transcribed by a professional transcriber and de-identified in order to retain the anonymity of participants and their counties.

**Table 1:** Interview guide for positive deviant counties

1	Let us start by having you describe what you do here. Would you tell us about your role related to promoting healthy living within the county/community?
2	Are residents or community leaders within your county concerned about obesity?
3	What particular strategies or initiatives of any kind are there that your county is implementing to prevent or reduce obesity rates (in children and/or adults)?
4	How does location/geography influence lifestyles in your county?
5	How would you describe the natural resources in particular?
6	What other types of resources are available in your county that you think affect obesity rates?
7	How would you describe the availability of the resources in the county?
8	How does your organization mobilize the resources, natural or other? How do you get the message out to the community that these resources are available?
9	How do you measure or report how many citizens within the county use the resources?
10	How do residents respond to and engage with these resources? Do they simply follow the programs, give active suggestions, inquire about ways they can help, go off to start their own initiatives etc.?
11	Are there activities you think are particularly effective at preventing or reducing obesity rates?
12	In terms of the programs/initiative that your organization is involved with what challenges have you faced in implementing them?
13	What elements (strategies/initiatives) has your organization implemented that proved to be the most successful over time? Are there any particular factors that you think influenced their sustainability?
14	Is there anything we haven’t discussed in terms of how your county is addressing the obesity epidemic that you think might be useful for us to know?

### Data analysis

We used the constant comparative method of qualitative data analysis [32, 35, 36]. A code sheet was developed by four research team members and then three members of the research team independently coded three randomly selected transcripts. Researchers assigned codes whenever a new concept was observed. Then the researchers met and collectively discussed the assigned codes and refined the code list. Two coders read each transcript independently and assigned codes to sections of text. The coders then held a series of meetings to reconcile their discrepancies and obtained a consensus for each code assignment. We used ATLAS.ti Scientific Software, version 7.1 to facilitate the organization and compilation of codes and retrieval of quotations to illustrate recurrent themes.

## Results

Our research aim was to identify positive deviant counties for obesity and using semi-structured in-depth interviews to determine any community level factors that were present across all counties that might give insights into how the lower rates of obesity were achieved by these counties. Several recurrent themes emerged from our analysis (described in detail below): 1) development of a nuanced understanding of their communities; 2) recognition of the complex nature of obesity, and 3) implementation of a county-wide strategic approach for promoting healthy living (Table 2).

**Table 2:** Key quotes from emergent themes

Theme	Quotation
Developing a nuanced understanding of their communities	*That was something that’s unique, I think, to this area, the trails and—because it is such a pretty area and then the lakes I think—which I don’t know that you could transfer that to another area, but I think the lakes bring a certain mindset about wanting to get out, and be in your swimsuit, and be on the water, and you’ve got to be somewhat physically fit to feel comfortable doing all that.*
	*This is just the way we’ve cooked for 20 years and it’s been handed down generation to generation. My great grandmother didn’t know that cooking in lard was not healthy, that’s all there was. It’s a new education for that generation saying, “Okay, you can still have this great food, but let’s try warming it up in the microwave instead of frying it in lard to warm it up.” There’s different methods.*
Recognizing the complex nature of obesity	*Overall, I think that the big take away… is giving people permission to say… my barriers may not be your barriers, and they might be emotional or they might be access or they might be economic or they might be education. Unfortunately, the problems are not easy, but we can facilitate cultures where people can find their own answers. When we do that, then I think you see success.*
	*We’re going to find out that it’s not just going to be any kind of food or physical activity, or any one, or even set of things necessarily focused on obesity or physical activity and nutrition, that’s creating a difference. I think you’re going to see it’s a real sweep of initiatives and social service movements that have combined.*
Developing a countywide strategic approach for promoting healthy living	*I think that I mentioned earlier the backbone organization, having a backbone organization that the community considers somewhat of a neutral convener. … I think at least in this community, what [we] brings to the table is a responsibility and a focus on the entire community… but having somebody, some organization who thinks of population broadly and is not necessarily a heavy provider of service. I’m not competing with anybody. I mean my job is to bring them all together and try to figure out how to help them work better together. It’s not to compete. I think that plays a big role and I think that you can’t convene something then expect it to run on its own. It takes tending and feeding and all of that to keep it moving forward.*
*Break down silos and build partnerships*	*I really, strongly believe that we’ve been working in our silo in the community on issues and we’ve been working in our silos in practices in medical community so they’re wanting us to improve health outcomes and reduce costs. If we keep working in our silos, although we might work a little bit differently, we’re not going have the impact we want. It’s got to be a comprehensive look.*
	*The goals are just different and you need to find that middle ground to where you can both say, “Okay, we’re both going to win from this,” and make the best of the situation and still find that audience that we’re looking for and get the message to them that we need to get to them.*
	*We have a AAA baseball park that just opened here recently. We were approached by them. We weren’t looking for the partnership, but they said, ‘We want to do what we can in our concessions to have healthy options for children. We heard about you guys. We want on board. What can we do?’ Not a conventional partnership, not something you would think about. Eating healthy is definitely not the ball park. You go to the ball park to eat junk and have fun and drink beer and watch a game.*
	*You just, I think, at a community level need to be open to unconventional partnerships and not be scared when somebody in the community approaches you about wanting to do something about obesity. You need to be willing to be flexible, and be adjustable, and think outside of the box on—it can’t be an automatic no. It needs to be, “Let’s sit back and think about this and think how we can work together.” It may not be something that’s traditional. It may be something off the wall and out of the box, but let’s try it and see what happens. I think some of our best partnerships have happened that way.*
*Tap into community resources and foster connections*	*The other part of [successful interventions] is we all [community leaders and community members] have to own it. It doesn’t belong to anyone in particular and as long as it doesn’t belong to anyone in particular and no one in particular is trying to take total credit for it and everybody is getting their share of the credit, that’s another reason for people to keep coming to the table.*
	*We spread ourselves a little thin for a while there and were doing so many different things that we couldn’t sustain that for too many years, and so kind of pulled back and started thinking more strategically and put a lot of efforts into, for example, this strategic plan that was designed to, okay, if we’re going to really do this over the long-term, let’s have some very clear goals for where we wanted to move towards.*
*Transferring ownership to the community members*	*We had a small number of extremely dedicated people…. We had these community advocates, and so those ringleaders, if you will, that were really critical for our efforts to be sustained over the longer term… Without that infrastructure, it just, at some point we just felt like we were spinning our wheels and no one had enough time to dig in and to say, “Okay, how do we prioritize the things that we need to do and how do we get the investment of, say, our city council and so forth?” Even with the very best of intentions and some pretty good energy, those kinds of efforts are really difficult to maintain over the longer term, and so it really does require an infrastructure that has some funding or that has the possibility.*
	*That’s the key, is to be able to measure and report out successes on a regular basis so that people are feeling like we’re making progress as long. As we’re making progress, people are going to be willing to keep coming to the table and the resources from the county for us to do this work will continue and the interest of funders of strategies that we’d like to do in the community will continue, but you have to be able to show the successes. I think that’s a key part to sustainability.*

Several participants described this county-wide strategic approach as building a “backbone” across their communities in order to tap into the county’s unique strengths and assets and counter the complex web of factors that leads to obesity. Participants described key elements of the process of building a county-wide backbone including: a) breaking down silos and building partnerships, b) accessing community resources and connections, and c) transferring ownership to community members.

- **Theme 1: Developing a nuanced understanding of their communities**
  Each of our participants described unique features of their counties that were important not only to community identity, but also as assets or liabilities in efforts to combat obesity and stimulate healthy living county-wide. The specific features highlighted by our participants varied, but the importance of developing a detailed understanding of factors specific to their communities emerged as a consistent theme across all interviews. As one example, a city finance accountant noted the importance of the natural landscape in their county:
*That was something that’s unique, I think, to this area, the trails and—because it is such a pretty area and then the lakes I think—which I don’t know that you could transfer that to another area, but I think the lakes bring a certain mindset about wanting to get out, and be in your swimsuit, and be on the water, and you’ve got to be somewhat physically fit to feel comfortable doing all that.*
  Others recognized the role of certain groups within their counties in shaping community identity. For example, some participants described large numbers of retirees, often transplants from elsewhere, whose imported views and expectations for healthy living had widespread influence on expectations and programs in the county.
  Respondents in this study also repeatedly described the potent relationship between poverty in their counties and obesity, recounting, for example, how some people would overeat unhealthy food (with their examples including fast food and whole milk in place of low-fat milk in schools) to offset a fear of food scarcity or insecurity. Additionally, participants described how struggles with poverty forced some community members to focus on meeting immediate needs, rather than making choices with long term health effects in mind. Together, these data indicated that developing a thorough and nuanced understanding of their communities was critically important in respondents’ efforts to counter obesity in a way that best aligned with the community members’ needs.
- **Theme 2: Recognizing the complex nature of obesity**
  Another consistent theme that emerged in our data was the importance of recognizing the complex web of factors that influence obesity. Participants noted that community members, including low-income parents trying to provide for their children as well as obese individuals approaching physical activity programs for the first time, experienced not just the physical elements of being obese but also psychological and emotional aspects. As one community leader described:
*Overall, I think that the big take away… is giving people permission to say… my barriers may not be your barriers, and they might be emotional or they might be access or they might be economic or they might be education. Unfortunately, the problems are not easy, but we can facilitate cultures where people can find their own answers. When we do that, then I think you see success.*
  Participants also consistently emphasized that obesity was a challenge to be considered at both the individual and community levels, and that addressing obesity at the county level means simultaneously addressing the needs and challenges of multiple communities with different dynamics.
*We’re going to find out that it’s not just going to be any kind of food or physical activity, or any one, or even set of things necessarily focused on obesity or physical activity and nutrition, that’s creating a difference. I think you’re going to see it’s a real sweep of initiatives and social service movements that have combined.*
  Additionally, the needs and challenges addressed by participants were finding alternative ways to provide and access services when public transportation was not available, such as offering bus passes and carpooling services or providing interventions within a local community to establish trust with community members. One health educator noted that prior to a low-income community being receptive to any health intervention program she met with leaders within the community several times and established a relationship with these individuals. An additional challenge noted by health educators was getting community members and leaders to see obesity as a multifaceted problem that required both physical and emotional investment and helping individuals overcome social and personal stigma associated with obesity.
- **Theme 3: Developing a countywide strategic approach for promoting healthy living**
  To address the complex web of factors that influence obesity in a way that best fit the unique features of their communities, participants described developing a countywide “backbone” to coordinate, synergize, and amplify the effectiveness of efforts to counter obesity within their boundaries. This backbone role was typically played by the county health department. One element of the backbone was logistical: bringing people together, facilitating their gathering, and enabling them to know about and talk to each other.
*I think that I mentioned earlier the backbone organization, having a backbone organization that the community considers somewhat of a neutral convener. … I think at least in this community, what [we] brings to the table is a responsibility and a focus on the entire community… but having somebody, some organization who thinks of population broadly and is not necessarily a heavy provider of service. I’m not competing with anybody. I mean my job is to bring them all together and try to figure out how to help them work better together. It’s not to compete. I think that plays a big role and I think that you can’t convene something then expect it to run on its own. It takes tending and feeding and all of that to keep it moving forward.*
  As described by our participants, the countywide backbone operated as a strategic approach to promoting healthy living through three main mechanisms: 3a) breaking down silos and building partnerships, both traditional and unconventional, 3b) tapping into community resources and connections, and 3c) transferring ownership to community members.
  - **Building a Backbone, Step 1: Break down silos and build partnerships**
    One of the main challenges that community leaders noted in attempting to counter the obesity epidemic was the longstanding existence of “silos,” or clear divisions and boundaries among different groups working in health and social services in their counties. Lack of connection among these silos led to a lack of coordination in programming to improve healthy eating and active living, as one health director described:
*I really, strongly believe that we’ve been working in our silo in the community on issues and we’ve been working in our silos in practices in medical community so they’re wanting us to improve health outcomes and reduce costs. If we keep working in our silos, although we might work a little bit differently, we’re not going [to] have the impact we want. It’s got to be a comprehensive look.*
    In order to be successful, community leaders noted that after breaking down the silos, they needed to build partnerships with different groups and organizations. Participants noted several strategies for bringing community members together, including having the county health department act as a neutral convener who would provide the physical meeting space and act as the logistical coordinator but then allow community members to lead meetings. In some communities, non-profit organizations rather than the county health department took on these convening and organizational tasks. Across communities, participants highlighted the importance of informal introductions by which the neutral convener would connect community leaders who had not previously collaborated but who were interested in the same types of interventions or populations. Such connections, viewed as valuable by participants, were often no more than personal introductions.
    Government officials often described more traditional partnerships with community organizations dedicated to nutrition or physical activity, such as parks and recreation departments, and they noted that a long history of collaboration contributed to trust and sustainability. Specific examples of these partnerships, seen in most or all of the positive deviant communities, involved school based nutrition education, physical activity programs, restaurants, and community gardens. In most counties, partnerships were also common with local universities to promote innovative interventions for different populations within a county including low-income communities. Collaborative efforts with local supermarkets for shopping tours and farmer’s markets were also a widely utilized partnership. But many of the participants also described working with a broader set of partners – sometimes quite unconventional, such as local minor league ballparks, former athletes or coaches, and refreshment providers at the zoo. Although the specific partner differed by county, health department staff across the counties noted the importance of instituting a “just say yes” strategy and that it was beneficial to meet with any groups that seriously approached them for collaboration as long as they could establish aligned goals for the population. Participants noted that employing a flexible, willing attitude in order to knit together these distinct but related elements of their communities was essential to improving the health of their community:
*You just, I think, at a community level need to be open to unconventional partnerships and not be scared when somebody in the community approaches you about wanting to do something about obesity. You need to be willing to be flexible, and be adjustable, and think outside of the box on—it can’t be an automatic no. It needs to be, “Let’s sit back and think about this and think how we can work together.” It may not be something that’s traditional. It may be something off the wall and out of the box, but let’s try it and see what happens. I think some of our best partnerships have happened that way.*
  - **Building a Backbone, Step 2: Tap into community resources and foster connections**
    Participants consistently emphasized that the health department, when functioning as a community-wide backbone, acted as both an organizer and convener for different organizations that promoted community health, to enable different partners to coalesce around a common, shared goal:
*The other part of [successful interventions] is we all [community leaders and community members] have to own it. It doesn’t belong to anyone in particular and as long as it doesn’t belong to anyone in particular and no one in particular is trying to take total credit for it and everybody is getting their share of the credit, that’s another reason for people to keep coming to the table.*
    Participants also noted that in order for any initiative to be successful, it was crucial to get broad, focused engagement across the county, which often meant facilitating community development of specific, strategic plans to combat obesity:
*We spread ourselves a little thin for a while there and were doing so many different things that we couldn’t sustain that for too many years, and so kind of pulled back and started thinking more strategically and put a lot of efforts into, for example, this strategic plan that was designed to, okay, if we’re going to really do this over the long-term, let’s have some very clear goals for where we wanted to move towards.*
  - **Building a Backbone, Step 3: Transferring ownership to the community members**
    The final element in establishing the county-level backbone for enacting successful interventions and strategies is sustainability. Participants described how programs that were dependent on funding and leadership from the state or federal government had limited shelf lives; instead, community members who joined healthy living initiatives were the true resources to maintain such efforts. Long-term success of programs or activities seemed most likely when both community leaders and community members took ownership and when specific champions supported these efforts.
    Participants also noted that demonstrating successes of programs and interventions helped sustain momentum and commitment. Such small victories helped show the community that time spent on such programs was worthwhile.
*That’s the key, is to be able to measure and report out successes on a regular basis so that people are feeling like we’re making progress as long. As we’re making progress, people are going to be willing to keep coming to the table and the resources from the county for us to do this work will continue and the interest of funders of strategies that we’d like to do in the community will continue, but you have to be able to show the successes. I think that’s a key part to sustainability.*

## Discussion

In six U.S. counties that are positive deviants for adult obesity rates, participants consistently described their efforts to develop a nuanced understanding of the features of their communities, while also recognizing that obesity was a complex challenge. They used this strategically gleaned knowledge to establish a countywide “backbone” structure that capitalized on strengths in their communities to combat obesity and improve healthy living. Although specific programs and initiatives differed across counties, participants consistently noted the importance of breaking down silos and building partnerships, fostering community connections, and transferring ownership to community members.

Study participants emphasized the importance of thinking broadly and holistically not only about the challenge of obesity but also about their approaches to address it. They were strategic in their planning and organization and deliberate in gathering information to develop tailored, countywide efforts to improve health and combat obesity. Although the group members who contributed to the backbone structure from across the county sometimes had different, individual goals, they came together with a common focus. Additionally, these individuals described an approach that reflected a systems-level view, thinking of themselves as contributing to an organizational structure that reaches across an entire complex system, pulling together different components (sometimes even those that are quite disparate from each other) in ways that introduced communications structures and capitalized on feedback loops, in service of the health of the whole [37–39].

Within the United States, obesity has often been approached as a medical problem to be handled at the individual level, often within the doctor-patient relationship. Obesity as a diagnosis was incorporated into the International Statistical Classification of Diseases and Related Health Problems (ICD) 10 [40]. Subsequently, many strategies to combat this epidemic propose an individual approach that is focused on losing body fat through diet or exercise or a combination of the two [15, 41]. Efforts to think beyond the individual level have focused more on organizations, such as schools or workplaces [22, 23], but little literature exists on countering obesity at the county level.

Findings from this study suggest that community-level, non-medical factors influence obesity and, further, that community-level strategies can be employed to counter this epidemic in the U.S. The strategies to address obesity described by study participants do not fit a medicalized, individual-level, disease-specific approach but instead focus on tapping into the unique strengths of a given community environment (e.g. natural resources, or an active retiree community). The community and government leaders we interviewed also emphasized the potential of tapping into and weaving together existing resources in a community, essentially helping to improve individual outcomes by focusing on social and community influences that surround and influence a given individual. Findings from this study suggest that successful strategies to counter obesity might need to extend beyond individual medical care and capitalize on resources in the community.

Our study has a variety of policy implications. Identifying and tapping into existing resources across a community may be a valuable strategy to combat the expensive challenge of obesity, especially if accompanied by investment in community-wide structures to convene, energize, and support strategic partnerships. Our findings suggest that for interventions to be sustainable, community members need to take initiative and ownership. However, health departments must have sufficient resources to play the key role of providing a backbone structure across the community.

Our study results should be interpreted in light of several limitations. First, we have a small sample of six counties. However, upon completing analyses of data from these counties, we reached theoretical saturation, and no new themes emerged. Second, as with any qualitative study, there is a risk of participant self-reporting bias; however, we used a variety of strategies as part of the interview process including open-ended questions and voluntary recording of the interview in order to promote an environment to encourage participants to speak candidly. Additionally, we conducted interviews with approximately 12 government and community leaders per county. It is possible that these participants might have had different perceptions of how their counties addressed the challenges of obesity than would other community members or participants. However, since this study is, to our knowledge, the first to apply the positive deviant approach to adult obesity rates at the county level, we chose government and community leaders as a crucial first group to interview. Finally, consistent with the positive deviant methodology [42–45], we focus on counties with the phenomenon of interest: relatively low obesity rates despite being located in a state with high obesity and having socioeconomic risk factors for high obesity. Such studies typically do not include comparison groups as those counties do not have the experience that is the focus of the inquiry. Nevertheless, as an extension of this exploratory, descriptive study, future investigations using a broader sample of counties with higher and lower rates of obesity and quantitative measurement of processes and structures would be helpful for testing the hypotheses generated from the present study.

## Conclusion

Obesity rates have steadily increased across the U.S. Our research suggests that positive deviant counties may provide a useful tool to learn about strategies and approaches that may be successful in fighting complex epidemics. Additionally, given the constraints from government resources including financial and human capital, communities can capitalize on members to promote and disseminate health messages and programs. Although fighting the challenge of obesity at a county-level is a daunting task; however, it can be aided by a focused approach that involves knowing the community and the complexity of health problems and incorporating that knowledge into the strategic plan.

## Abbreviations

BMI: Body mass index
ICD: International Statistical Classification of Diseases and Related Health Problems
